# Epidemiological and Clinical Features of Hepatitis B Virus Genotypes among Immigrants in Southern Italy

**DOI:** 10.1155/2010/878356

**Published:** 2010-04-22

**Authors:** Gaetano Scotto, Domenico Martinelli, Rocco Di Tullio, Vincenzina Fazio

**Affiliations:** ^1^Clinica di Malattie Infettive, Università degli Studi di Foggia, Foggia 71100, Italy; ^2^Sezione di Igiene, Dipartimento di Scienze Mediche ed Occupazionali, Università di Foggia, Foggia 71100, Italy; ^3^Dipartimento di Laboratorio, Ospedali Riuniti, Foggia 71100, Italy

## Abstract

*Background/aims*. This study aims to determine the distribution and clinical features of HBV-genotypes in a population of immigrants affected by HBV-infection. *Methods*. Between 01/2003 and 03/2009, 1623 immigrants were tested for HBV-infection. Biochemical and virological activities were determined in HBsAg-positive patients; HBV-genotypes were determined, by the INNO-LiPA HBV Genotyping, in the subjects with HBV DNA detectable. In every patient we evaluated the stage and classified the infection as inactive carrier, mild or moderate/severe chronic hepatitis, cirrhosis, and/or HCC. *Results*. Among the tested subjects, 191 (11.7%) resulted HBsAg-positive, and in 144/191 (75.4%) serum HBV-DNA was detectable. The genotype distribution was as follows: 45,13% genotype E, 18,1% genotype D, 15,3% genotype B, 13,2% genotype C, 4,9% genotype A, 3,5% mixed genotypes (A–D). The evaluation of liver disease degree showed that 24.6% patients were inactive carriers of HBV infection, 19.4% presented a immunotolerance phase, 34.5% had mild chronic hepatitis, 13.6% had a moderate/severe chronic hepatitis, 6.3% had cirrhosis, and 1.6% presented HCC. *Conclusions*. Our study evidences a high prevalence of HBV-infection in immigrants, and the potentiality of migratory flow in the introduction of genotype non-D hepatitis B virus. The Hepatitis B virus genotypes presented significant differences in epidemiological and clinical characteristics.

## 1. Introduction

Hepatitis B virus (HBV) infection is a global health problem, approximately 2 billion people in the world have been infected by HBV, and more than 350 million are chronic carriers of the virus. This infection is present mainly in some countries as Middle-East and South-East Asia, sub-Saharan Africa, Central and South-America, and East-Europe with a prevalence >8% of population [1]. In the last twenty years a migratory flow has been going on from these countries to the industrialized countries (USA and West-Europe) with an increase of some infectious diseases (HIV, TB, viral hepatitis) [2, 3].

 The migratory flow towards Italy has considerably increased in the last 10 years: at present the total number of legal immigrants can be estimated as 3.700.000 subjects (6.2% of all population) [4]. When considering these figures, it is obvious that, apart from questions regarding human rights, social-economic, and cultural integration, the range of health problems in immigrants is inevitably broad and includes, mainly, infectious diseases.

 In our precedent study, the acute or chronic viral hepatitis represented the third cause of hospitalization for infectious diseases in immigrants (12% of cases) after HIV infection (16%) and tuberculosis (13%). The viral hepatitis is most frequently related to HBV, particularly in African people coming from sub-Saharan areas, and South-East, or Far-East Asians [5].

 But HBV infection is not always the same that we have been knowing for years in Italy, it is often a new infection with virological and clinical characteristics, HBV-genotypes related. 

 The HBV genome is a partially double-stranded circular DNA molecule consisting of 3200 nucleotides and exhibits considerable genetic variability; based on a nucleotide divergence >8% in the entire viral genome, HBV has been established and classified into eight genotypes (A to H) with a characteristic geographic distribution. Genotype A is widely distributed in North-West Europe, North-America and Central Africa, while genotype B and C are present in Asia only; genotype D has been found worldwide with its highest prevalence in the Mediterranean area, the Middle East, and South-Asia, particularly in India; genotype E is found in Sub-Saharan Africa and genotype F in South and Central America; genotype G has been found in France and in the USA, while the newly discovered genotype H seems so far to be restricted to the northern part of Latin America, including Central America and Mexico [6, 7].

 The accumulated evidences suggest that HBV genotypes have an impact on the natural course of chronic HBV infection with differences in the severity of underlying liver disease or treatment response [8–10].

 In Italy, about 95% of Italian patients with HBV infection presents the genotype D [6]. Since the migratory flow towards our country is coming from endemic areas for HBV (prevalence >8%) such as East-Europe, sub-Saharan Africa and East Asia, the introduction in Italy of nonD HBV genotypes infection could be a natural consequence. This may be important both from the epidemiological and clinical point of view. In immigrants who live in different districts of Southern Italy the genotype distribution is strictly related to the typology of immigration and reflects the distribution for the areas of origin. In Campania and Lucania immigrants are coming mainly from Middle East Asia and North Africa; in Sicilia and Calabria from Middle East Asia, North Africa and from Central Africa; in Puglia the immigration is mainly from East Europe and from sub-Saharan Africa. In our geographical area (Foggia) there are mainly immigrants coming from Sub-Saharan Africa, East Europe and Far-East Asia. The aim of the present study is to determine, in a population of recent (<6 months) immigrants the geographic distribution and virological characteristics of HBV genotypes and the association between different HBV genotypes and the clinical status of patients.

## 2. Materials and Methods

Between January 2003 and March 2009 a total of 1623 volunteer immigrants (about 45% of total population) were tested for infectious screening (HIV-HCV-TPHA-TB and HBsAg-HBsAb-HBcAb of HBV) after obtaining appropriate informed consent. All tested subjects were temporarily guests in a refugee camp managed by the Italian Red Cross, located in Borgo Mezzanone (province of Foggia, Italy).

 The study was reviewed and approved by the local Chief of the Red Cross and written informed consent was obtained from each study subject who was enrolled in the study. All study procedures were in agreement with the Helsinki Declaration (Edinburgh 2000). At enrolment, all study participants were interviewed using a questionnaire to obtain baseline demographic, clinical, and socioeconomic information and to assess their previous exposure to HBV Infection. All enrolled subjects also received a full clinical examination and were treated accordingly.

 The average length of staying in Italy was 2 months (range 13–57 days) and the majority (1286, 79.3%) came from Africa (41% Eritrea, 35% Somalia, 7.7% Liberia); 239 subjects (14.7%) came from Asia (8.8% Pakistan, 3.2% China, 0.5% Iraq), while 98 subjects (6.0%) came from East-Europe (3.8% Romania, 2.2% Albania).

 The mean age of the tested subjects was 27.3 years (range 1–56) and most of them were men (83.7%). We evaluated, in HBsAg positive individuals, the biochemical and virological activity of infection and the eventual presence of coinfections (HCV, HDV, HIV); the HBVgenotype was determined in those with detectable serum HBV-DNA. HBsAg was assayed by commercial immunoassay (Abbott-Auszyme Mc, Abbott Laboratories, North Chicago, IL, USA). Hepatitis B E Antigen (HbeAg) and antibody to HBeAg (anti-HBe) were detected by radioimmunoassay (HBeAg/antiHBe immunoradiometric DiaSorin, Vercelli, Italy). IgM and IgG anti-HDV were tested with commercially available ELISA kits (Abbott Diagnostics, Weisbaden-Delkenheim, Germany). The presence of antibodies to HCV (anti-HCV) was determined by the use of a third-generation enzyme-linked-immunoabsorbent assay (HCV-ELISA, Ortho Diagnostic System, Raritan, NJ, USA) and confirmed by a third-generation-recombinant-immunoblot assay (RIBA, Ortho Diagnostic Systems, Raritan, NJ, USA). Antibodies to HIV (anti-HIV) were determined by EIA (HIV1/HIV2, Abbott) and positive results were confirmed by Western Blot. Serum HBV-DNA levels were measured by a commercial PCR-real time with a detection limit of 100 copies/mL. Serum ALT was quantified by ultraviolet-enzymatic-assay (normal range 0–40 IU/L). For the determination of HBV genotypes, HBV-DNA was extracted as described by Stuyver et al. [11]. The extracted DNA was amplified over two rounds of PCR using biotinylated PCR primers. After the two PCR for amplification, HBV-genotypes were determined by the INNO-LiPA HBV Genotyping (Innogenetics NV, Gent, Belgium). The correct HBV genotype was determined by consulting an interpretation chart showing probe reactivity patterns for each HBV genotype.

## 3. Evaluation of Liver Disease Degree

All the patients with biochemical and/or virological activity of HBV infection refused to undergo liver biopsy. Therefore, for the evaluation and classification of liver disease degree we considered several parameters: bilirubine, AST/ALT (ratio >1 or <1 in nonalcoholic patients), viral load (<10^5^ or >10^5^ copies/mL), count of platelets (>10^5^/mm^3^ or <10^5^/mm^3^), plasma proteins (hypoalbuminaemia, increased gammaglobulins), apolipoproteine A_1_, aptoglobine, *α*2-macroglobuline, cholesterol, prothrombine time, gamma-glutammil-transferase, pseudocholinesterasis, abdominal echography. The disease degree was based on the analysis of more variable parameters; only for hepatocellular carcinoma (HCC) the main examen was the abdominal echography, followed, after the first diagnosis, by alpha-phetoprotein and abdominal TC. 

The different degrees of chronic hepatitis B can be schematically divided into the following.

1. “Immunotolerance phase” is characterized by HBeAg positivity (wild type infection), high levels of HBV replication (reflected by high levels of serum HBV DNA), normal or low levels of aminotransferase, mild or no liver necroinflammation and no or slow progression of fibrosis.
2. Inactive HBV carrier state may follow seroconversion from HBeAg to anti-HBe antibodies. It is characterized by very low or undetectable serum HBV DNA levels and normal aminotransferase.
3. Mild hepatitis: is marked by expansion of the portal zone by mononuclear cells and some fibrosis. The limiting plate of liver cells between portal zones and liver cell column is intact. Piecemeal necrosis of liver cells is not seen.
4. Moderate hepatitis is marked by the presence of an inflammatory infiltrate, primarily of llymphocytes and plasma cells, which greatly expands the portal areas. The inflammatory infiltrate extends into the liver lobule, causing erosion of the limiting plate and piecemeal necrosis.
5. Severe hepatitisis marked by fibrous septa extending into the liver columns with isolation of groups of liver cells in the form of rosettes. Intrahepatic “bridging”, either portal-central or portal-portal, is seen.

## 4. Statistical Analysis Methods

To analyze the associations between genotypes (A, B, C, D, E, and A-D mixed) and countries of origins, transaminases levels (AST and ALT >40 UI/mL), and stage of disease (immunotollerance, mild hepatitis, moderate/severe hepatitis, cirrhosis, hepatocellular carcinoma), 2 × 2 contingence tables were constructed and chi square (*χ* ^2^) or Fisher test was calculated. When it was possible, OR and its relative 95% CI was calculated. To compare the levels of viremia among different genotypes, Kruskal-Wallis test was performed. The chosen level of statistical significance was *P* < .05. Statistical calculations were performed using STATA 10 MP for Mac Os.

## 5. Results

Among the 1623 tested subjects, 191 (11.7%) resulted HBsAg positive, 146 males and 45 females. The mean age of positive subjects was 26 years (range 19–47); 96 patients (50.2%) came from Africa (30 from Liberia, 42 from Eritrea, and 24 from Somalia), 59 (31.0%) from Asia (38 from China and 21 from Pakistan) and 36 (18.8%) from East-Europe (19 from Romania and 17 from Albania). One hundred and forty-eight patients were anti-HBe positive while 43 presented HBV wild (HBsAg-HBeAg positive) type. No subject presented coinfection with HIV. Sixteen patients showed coinfection with HDV, nine with HCV and three with HCV-HDV. All the subjects with coinfection did not present HCV and/or HDV viral replication.

Forty seven/191 (24.6%) patients presented normal ALT levels (<40 IU/L) and undetectable serum HBV DNA (<100 copies/mL), thirty-seven (19.4%) subjects presented persistent normal ALT levels, but HBV-DNA detectable by PCR Real Time (mean level 1374231 copies/mL, range 74651–13.875.247), while 107 (56.0%) patients had elevated ALT levels (mean level 141 IU/L, range 68–297) and serum HBV-DNA detectable (mean level 1561183 copies/mL, range 22.933–11.290.865).

 Genotype distribution, among all the HBV DNA positive patients, was determined as follows: 65 genotype E (45.13%), 26 genotype D (18.1%), 22 genotype B (15.3%), 19 genotype C (13.2%), 7 genotype A (4.9%), and 5 mixed genotypes (A–D) (3.5%). Genotype E was associated to Eritrean (OR: 26.1, 95% CI: 7.2–139.2; *χ* ^2^ = 41.96, *P* < .001) and Liberian subgroups (OR and 95% CI not calculable; *χ* ^2^ = 31.56, *P* < .001). Genotype A was associated to Somali subgroup (OR: 16.9, 95% CI: 2.4–126.5; *χ* ^2^ = 18.85, *P* < .001). Genotypes C and B were associated to Chinese subgroup (resp., OR: 39.1, 95% CI: 9.3–223.4; *χ* ^2^ = 50.9, *P* < .001 (OR: 14.1, 95% CI: 4.5–46.7; *χ* ^2^ = 33.46, *P* < .001). Genotype D was associated to Pakistani (OR: 15.4, 95% CI: 5.2–47.6; *χ* ^2^ = 38.53, *P* < .001) and to Albania subgroups (OR and 95% CI: not calculable; *χ* ^2^ = 38.44, *P* < .001). Also genotypes A–D mixed was associated to Pakistani subgroup (OR and 95% CI: not calculable; *χ* ^2^ = 17.42, *P* < .001). 

 HBV wild type infection was present in 15/19 patients with genotype C (78.9%), in 14/22 patients with genotype B (63.6%), in 12/65 patients with genotype E (18.5%), and in 2/26 patients with genotype D (7.7%). 

 The mean ALT, in the patients with altered serum level, was similar among the five different genotypes (143 ± 37 UI/mL.), without any association between any genotypes and AST and ALT > 40 UI/mL (*P* > .05), respectively. The level of HBV replication differed between HBV genotypes, with a mean rate of 1.5 × 10^6^ copies/mL (range 2.2 × 10^4^–1.1 × 10^7^), with the highest values for genotype C and the lowest meanvalues for genotype E (8.7 × 10^5^ copies/mL). There was a significant difference in levels of viremia among different genotypes (*χ* ^2^ = 54.47, *P* < .001) (Table 1).

 The evaluation of liver disease degree showed that 47/191 (24.6%) patients were inactive carriers of HBV infection, 37 presented a phase of immunotolerance, 66 (34.5%) had mild chronic hepatitis, 26 (13.6%) had a moderate/severe chronic hepatitis, 12/191 (6.3%) had cirrhosis and 3 (1.6%) presented HCC. In Table 2 is shown the HBV infection degree according to HBV genotypes.

 There was not association between any genotypes and immunotollerance state (*P* > .05). Genotype E was associated with “mild chronic hepatitis” (OR: 4.2, 95% CI: 2–8.9; *χ* ^2^ = 16.84, *P* < .001). There was not association between any genotypes and “moderate/severe hepatitis or cirrhosis” (*P* > .05). Genotypes A–D mixed was associated to “hepatocellular carcinoma” (OR: 92, 95% CI: 3.2–5455.3; *P* < .05). 

 Looking at the thirty-eight patients with moderate/severe hepatitis or cirrhosis, in 20 of them (52.6%) the disease was related to HBV wild infection, while 18 (47.4%) were anti-HBe positive (*P* > .05).

## 6. Discussion

Some recent studies have shown how in immigrants HBV infection represents one of the prevalent infectious diseases, particularly in African people coming from sub-Saharan areas [5, 7, 12, 13], with a rate of infection >8%, higher than in Western Countries, probably caused by the lack or incomplete prophylactic vaccination in the country of origin and with risky sexual behaviour. 

 In our study we evaluated the prevalence, the typology of HBV-infection and the degree of disease, according to genotypes, in a population of immigrants to Italy, by a mean period of 2 months. This is the first study in Italy which evaluates the stage of HBV chronic hepatitis according to its viral genotype; previous studies had only showed the heterogeneity of the virus according to the subjects' geographical area [14, 15]. The results (HBsAg positive rate of 11.7%) reflect the actual prevalent migratory flow to Italy, especially in our geographic area, mostly represented by people coming from Africa [4]. An important finding was that almost all HBsAg-positive subjects were men, but this data could be related to the difficulty in testing females (only 349/1623 of tested subjects were females). In fact, in African communities females fear to know their eventual diseases, particularly infectious diseases which are considered a further cause of social discrimination [5].

 The vast majority of cases were infected by genotype E and this coincides with the usual geographic distribution of this genotype, which is exclusively diffused in sub-Saharan areas [13]. Other HBV genotypes were also found among immigrants to our country, like D-B-C-A and mixed genotypes, thus reflecting different areas of origin [11, 16, 17]. This redistribution of HBV genotypes represent an important change in the epidemiology of infection, increasing the number of infected subjects in our country, with a “different” hepatitis, also if the risk of contamination with a “new virus” for Italian population is low, because the HBV vaccination defends from a reinfection.

 Recently, there have been several studies reporting the influence of HBV genotypes on the clinical features [8, 9, 18] and on the response to antiviral treatment (interferon and lamivudine) of patients infected with HBV [10, 19, 20]. Therefore the different genotypes, probably characterized by a different natural history and a different response to therapy, could require a different clinical and therapeutic approach compared to genotype-D. In order to further investigate these associations, we have examined in our patients the influence of HBV genotypes on the liver disease progression. All the patients refused to undergo liver biopsy, therefore for the evaluation and classification of liver disease degree we considered the noninvasive markers of fibrosis and inflammation in clinical practice, mainly the Fibrotest/Apritest, because the data of literature showed that these markers are a reliable method for predicting significant liver fibrosis and necroinflammation in both viral and nonviral chronic liver disease patients. 

 Several tested subjects (about 25%) were inactive HBV carriers. A normal ALT level finding is not surprising, since HBV-infection has an high prevalence (>8%) in sub-Saharan people, but many infected subjects (a variable rate of 40%–65% in different studies) do not show disease's biochemical activity [13]. 

 The prevalence of immunotolerance phase and mild chronic hepatitis was mainly observed in patients with genotype E. This is absolutely usual given the origin of the patients and the fact that at least part of them (and probably not a small one) is supposed to have acquired HBV vertically and thus might have developed immunotolerance to the virus. These ones were all from Sub-Saharan Africa, where HBV transmission mostly occurs during early childhood, and were characterized by both a younger age and a lower viral load than the other genotypes. Despite high endemicity, little was known about HBV genotype distribution across Africa until recently, when genotype E was found to be characterized by a high prevalence and vast geographical distribution (about one-third) in the African continent [21].

 In a study among blood donors in Ghana, all infected by genotype E, the mean viral load was relatively low even in 16–19-years-old patients. Therefore the highest rate of mild disease in genotype E, observed in our study, may be related overall to younger age of infection and lower HBV-DNA levels [22]. 

 In conclusion, our study evidences a high prevalence of HBV-infection in immigrants. The global migratory flow to our country, mainly from tropical areas, can determine a partial modification of the normal geographic distribution of HBV-genotypes, with the introduction of nonD genotypes, potentially characterized by different natural history and response to antiviral regimens.

## Figures and Tables

**Table 1:** Distribution of HBV genotypes according to country of origin, mean of transaminase (*P* > .05), and mean of viremia (*P* < .001).

Genotypes	Patients	Country of origin	Mean AST/ALT	Mean HBV-DNA
			levels (nv < 40 UI/L)	(nv < 20 copies/mL)
E	**65** (45,13%)	33 Eritrea	124/150	871.900
		22 Liberia		
		10 Somalia		

D	**26** (18,1%)	10 Romania	131/137	1.278.831
		8 Albania		
		8 Pakistan		

B	**22** (15,3%)	15 China	132/143	1.176.900
		7 Pakistan		

C	**19** (13,2%)	16 China	118/154	2.961.000
		3 Pakistan		

A	**7** (4,9%)	4 Somalia	112/128	1.343.272
		3 Eritrea		

MIxed (A/D)	**5** (3,5%)	5 Romania	127/135	1.735.200

**Table 2:** HBV infection degree according to genotype, see the text.

	Genotypes					
	E	D	B	C	A	Mixed
Immunotolerance *P* > .05	16	7	9	3	2	0
Mild chronic hepatitis	42	9	7	6	2	0
	*P* < .001					
Moderate/severe chron. hepatitis *P* > .05	7	6	4	5	2	2
Cirrhosis *P* > .05	0	4	2	4	1	1
Hepatocarcinoma						2
Mixed *P* < .05	0	0	0	1	0	
